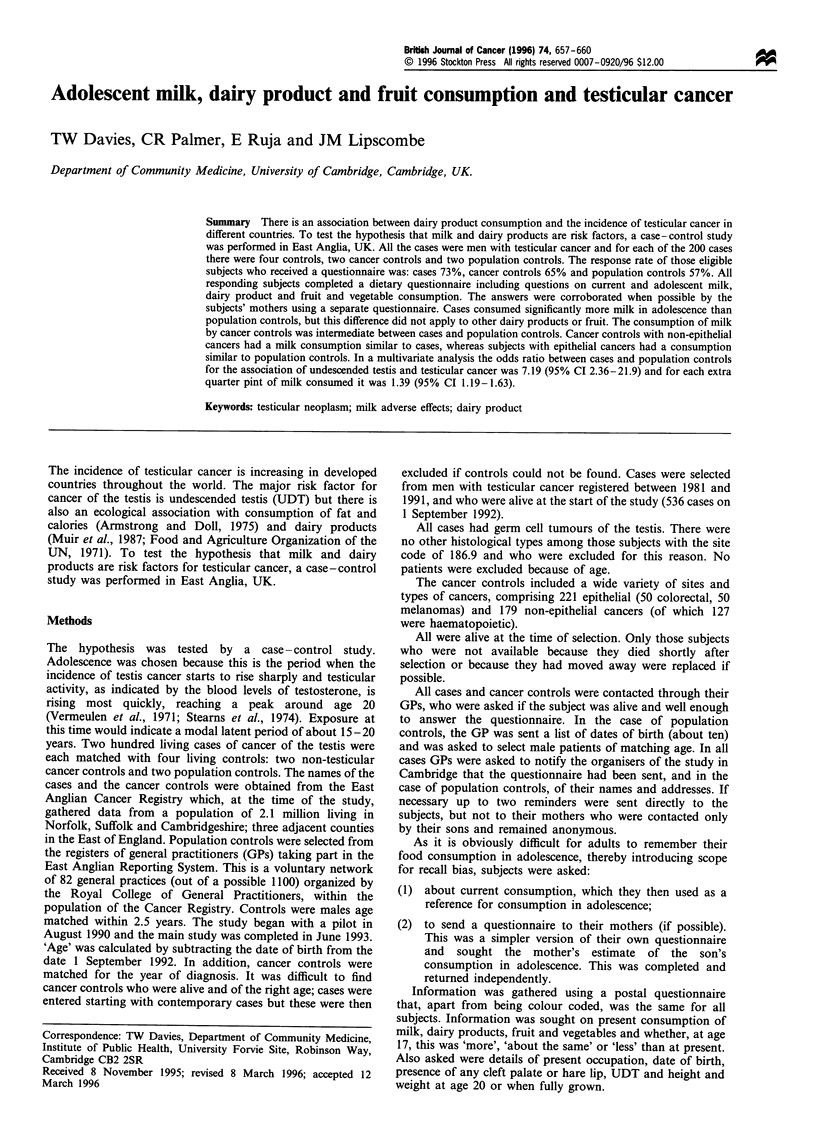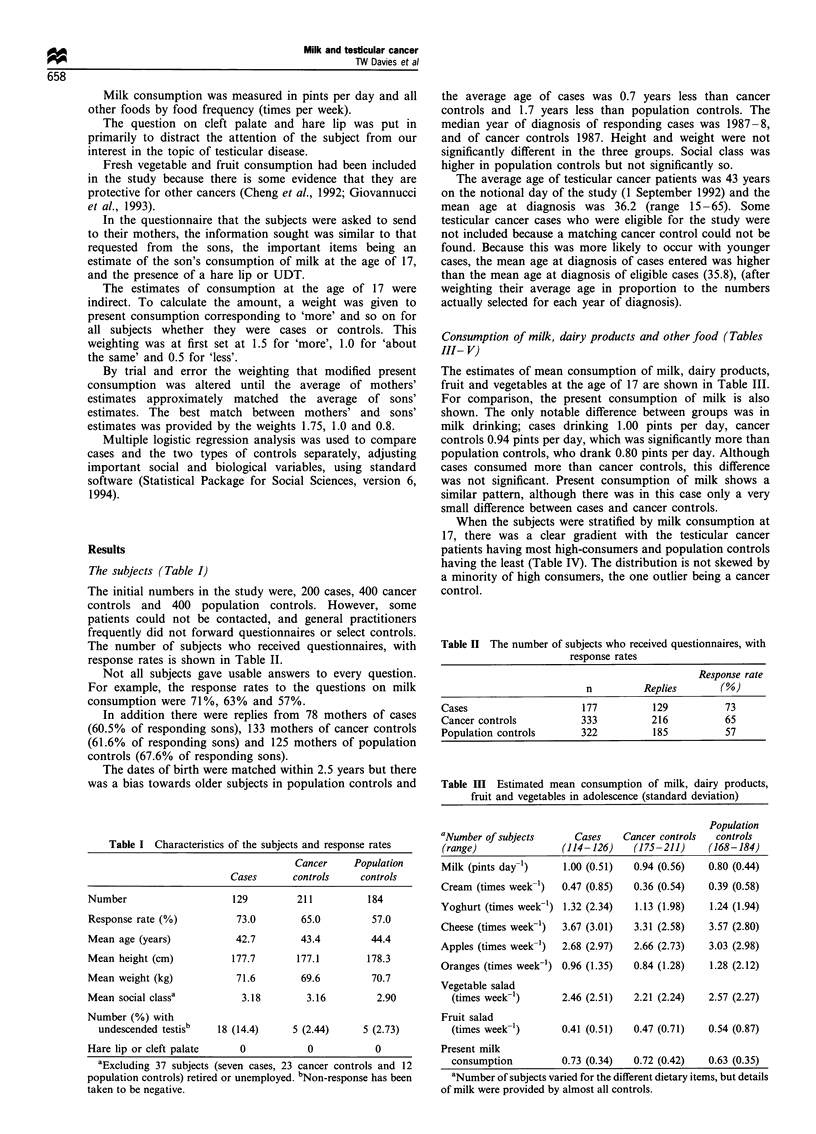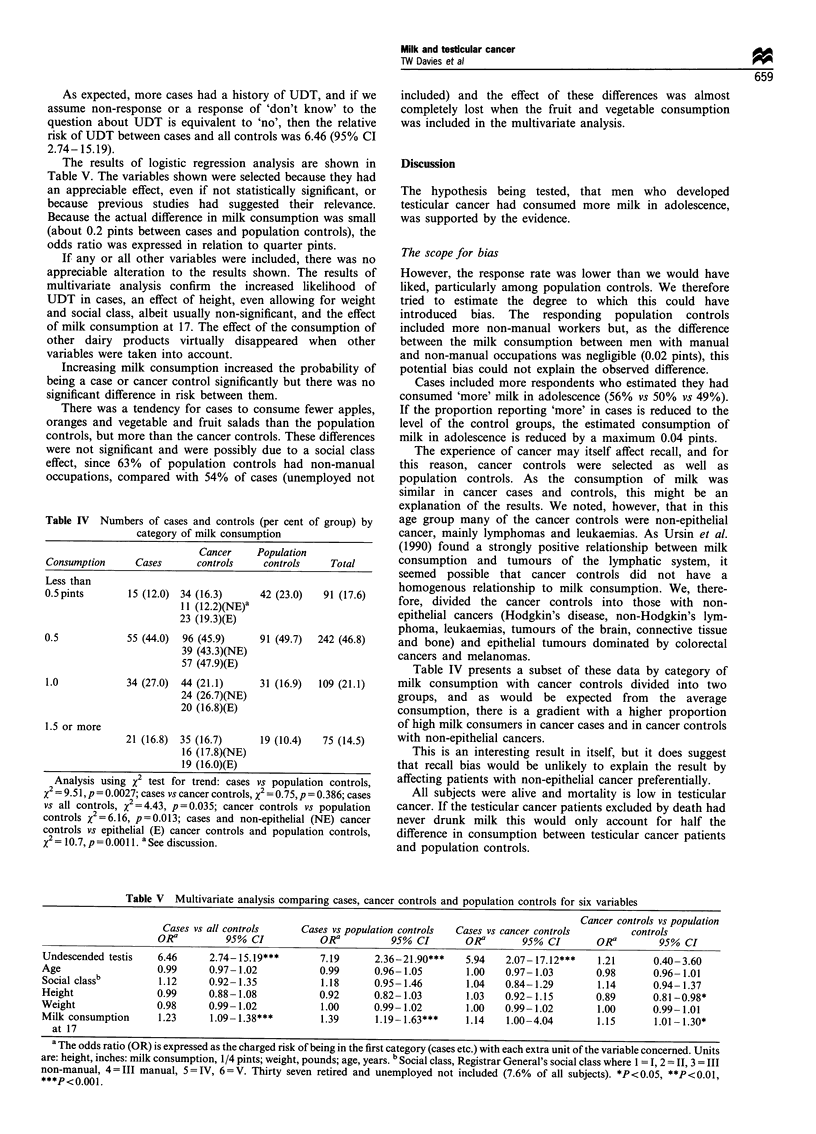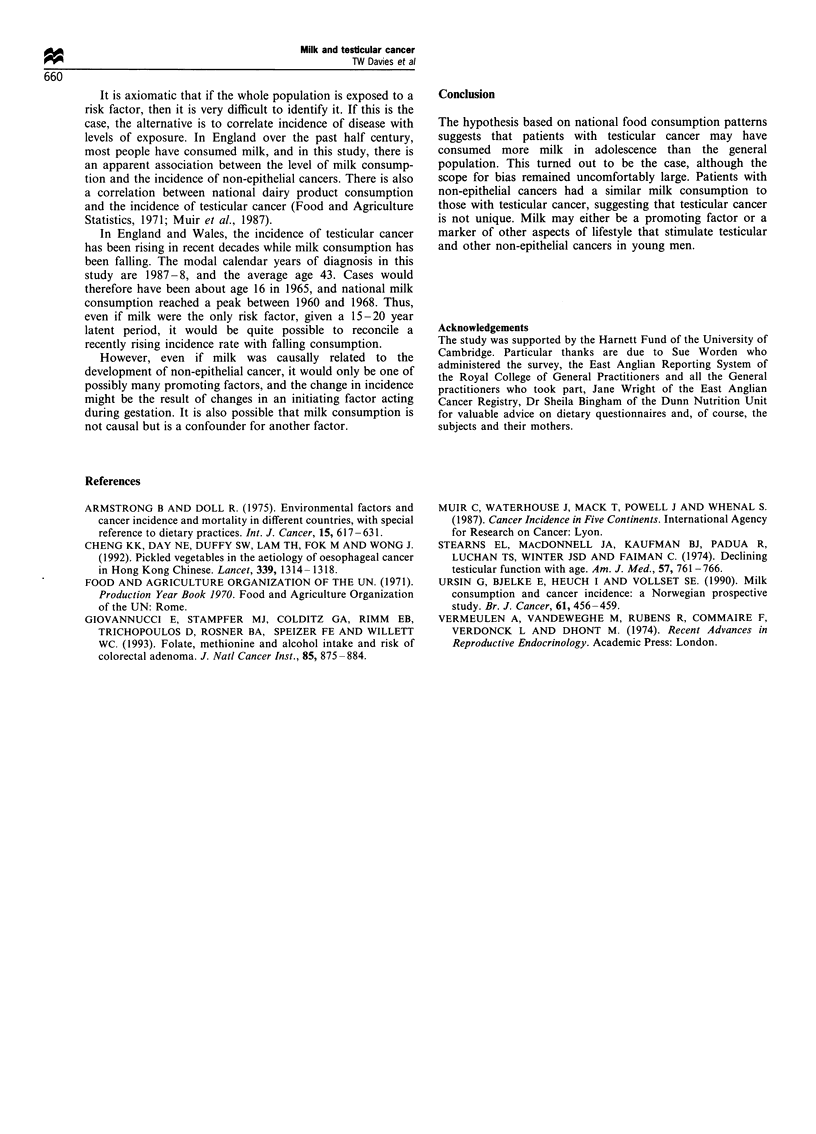# Adolescent milk, dairy product and fruit consumption and testicular cancer.

**DOI:** 10.1038/bjc.1996.417

**Published:** 1996-08

**Authors:** T. W. Davies, C. R. Palmer, E. Ruja, J. M. Lipscombe

**Affiliations:** Department of Community Medicine, University of Cambridge, UK.

## Abstract

There is an association between dairy product consumption and the incidence of testicular cancer in different countries. To test the hypothesis that milk and dairy products are risk factors, a case-control study was performed in East Anglia, UK. All the cases were men with testicular cancer and for each of the 200 cases there were four controls, two cancer controls and two population controls. The response rate of those eligible subjects who received a questionnaire was: cases 73%, cancer controls 65% and population controls 57%. All responding subjects completed a dietary questionnaire including questions on current and adolescent milk, dairy product and fruit and vegetable consumption. The answers were corroborated when possible by the subjects' mothers using a separate questionnaire. Cases consumed significantly more milk in adolescence than population controls, but this difference did not apply to other dairy products or fruit. The consumption of milk by cancer controls was intermediate between cases and population controls. Cancer controls with non-epithelial cancers had a milk consumption similar to cases, whereas subjects with epithelial cancers had a consumption similar to population controls. In a multivariate analysis the odds ratio between cases and population controls for the association of undescended testis and testicular cancer was 7.19 (95% CI 2.36-21.9) and for each extra quarter pint of milk consumed it was 1.39 (95% CI 1.19-1.63).


British Journal of Cancer (1996) 74, 657-660
© 1996 Stockton Press All rights reserved 0007-0920/96 $12.00

### Adolescent milk, dairy product and fruit consumption and testicular cancer

TW Davies, CR Palmer, E Ruja and JM Lipscombe

*Department of Community Medicine, University of Cambridge, Cambridge, UK.*

**Summary** There is an association between dairy product consumption and the incidence of testicular cancer in different countries. To test the hypothesis that milk and dairy products are risk factors, a case–control study was performed in East Anglia, UK. All the cases were men with testicular cancer and for each of the 200 cases there were four controls, two cancer controls and two population controls. The response rate of those eligible subjects who received a questionnaire was: cases 73%, cancer controls 65% and population controls 57%. All responding subjects completed a dietary questionnaire including questions on current and adolescent milk, dairy product and fruit and vegetable consumption. The answers were corroborated when possible by the subjects' mothers using a separate questionnaire. Cases consumed significantly more milk in adolescence than population controls, but this difference did not apply to other dairy products or fruit. The consumption of milk by cancer controls was intermediate between cases and population controls. Cancer controls with non-epithelial cancers had a milk consumption similar to cases, whereas subjects with epithelial cancers had a consumption similar to population controls. In a multivariate analysis the odds ratio between cases and population controls for the association of undescended testis and testicular cancer was 7.19 (95% CI 2.36–21.9) and for each extra quarter pint of milk consumed it was 1.39 (95% CI 1.19–1.63).

**Keywords:** testicular neoplasm; milk adverse effects; dairy product

The incidence of testicular cancer is increasing in developed countries throughout the world. The major risk factor for cancer of the testis is undescended testis (UDT) but there is also an ecological association with consumption of fat and calories (Armstrong and Doll, 1975) and dairy products (Muir *et al.*, 1987; Food and Agriculture Organization of the UN, 1971). To test the hypothesis that milk and dairy products are risk factors for testicular cancer, a case–control study was performed in East Anglia, UK.

### Methods

The hypothesis was tested by a case–control study. Adolescence was chosen because this is the period when the incidence of testis cancer starts to rise sharply and testicular activity, as indicated by the blood levels of testosterone, is rising most quickly, reaching a peak around age 20 (Vermeulen *et al.*, 1971; Stearns *et al.*, 1974). Exposure at this time would indicate a modal latent period of about 15–20 years. Two hundred living cases of cancer of the testis were each matched with four living controls: two non-testicular cancer controls and two population controls. The names of the cases and the cancer controls were obtained from the East Anglian Cancer Registry which, at the time of the study, gathered data from a population of 2.1 million living in Norfolk, Suffolk and Cambridgeshire; three adjacent counties in the East of England. Population controls were selected from the registers of general practitioners (GPs) taking part in the East Anglian Reporting System. This is a voluntary network of 82 general practices (out of a possible 1100) organized by the Royal College of General Practitioners, within the population of the Cancer Registry. Controls were males age matched within 2.5 years. The study began with a pilot in August 1990 and the main study was completed in June 1993. 'Age' was calculated by subtracting the date of birth from the date 1 September 1992. In addition, cancer controls were matched for the year of diagnosis. It was difficult to find cancer controls who were alive and of the right age; cases were entered starting with contemporary cases but these were then excluded if controls could not be found. Cases were selected from men with testicular cancer registered between 1981 and 1991, and who were alive at the start of the study (536 cases on 1 September 1992).

All cases had germ cell tumours of the testis. There were no other histological types among those subjects with the site code of 186.9 and who were excluded for this reason. No patients were excluded because of age.

The cancer controls included a wide variety of sites and types of cancers, comprising 221 epithelial (50 colorectal, 50 melanomas) and 179 non-epithelial cancers (of which 127 were haematopoietic).

All were alive at the time of selection. Only those subjects who were not available because they died shortly after selection or because they had moved away were replaced if possible.

All cases and cancer controls were contacted through their GPs, who were asked if the subject was alive and well enough to answer the questionnaire. In the case of population controls, the GP was sent a list of dates of birth (about ten) and was asked to select male patients of matching age. In all cases GPs were asked to notify the organisers of the study in Cambridge that the questionnaire had been sent, and in the case of population controls, of their names and addresses. If necessary up to two reminders were sent directly to the subjects, but not to their mothers who were contacted only by their sons and remained anonymous.

As it is obviously difficult for adults to remember their food consumption in adolescence, thereby introducing scope for recall bias, subjects were asked:

(1) about current consumption, which they then used as a reference for consumption in adolescence;

(2) to send a questionnaire to their mothers (if possible). This was a simpler version of their own questionnaire and sought the mother's estimate of the son's consumption in adolescence. This was completed and returned independently.

Information was gathered using a postal questionnaire that, apart from being colour coded, was the same for all subjects. Information was sought on present consumption of milk, dairy products, fruit and vegetables and whether, at age 17, this was 'more', 'about the same' or 'less' than at present. Also asked were details of present occupation, date of birth, presence of any cleft palate or hare lip, UDT and height and weight at age 20 or when fully grown.

Correspondence: TW Davies, Department of Community Medicine, Institute of Public Health, University Forvie Site, Robinson Way, Cambridge CB2 2SR
Received 8 November 1995; revised 8 March 1996; accepted 12 March 1996

Milk consumption was measured in pints per day and all other foods by food frequency (times per week).

The question on cleft palate and hare lip was put in primarily to distract the attention of the subject from our interest in the topic of testicular disease.

Fresh vegetable and fruit consumption had been included in the study because there is some evidence that they are protective for other cancers (Cheng *et al.*, 1992; Giovannucci *et al.*, 1993).

In the questionnaire that the subjects were asked to send to their mothers, the information sought was similar to that requested from the sons, the important items being an estimate of the son's consumption of milk at the age of 17, and the presence of a hare lip or UDT.

The estimates of consumption at the age of 17 were indirect. To calculate the amount, a weight was given to present consumption corresponding to 'more' and so on for all subjects whether they were cases or controls. This weighting was at first set at 1.5 for 'more', 1.0 for 'about the same' and 0.5 for 'less'.

By trial and error the weighting that modified present consumption was altered until the average of mothers' estimates approximately matched the average of sons' estimates. The best match between mothers' and sons' estimates was provided by the weights 1.75, 1.0 and 0.8.

Multiple logistic regression analysis was used to compare cases and the two types of controls separately, adjusting important social and biological variables, using standard software (Statistical Package for Social Sciences, version 6, 1994).

### Results

### *The subjects (Table I)*

The initial numbers in the study were, 200 cases, 400 cancer controls and 400 population controls. However, some patients could not be contacted, and general practitioners frequently did not forward questionnaires or select controls. The number of subjects who received questionnaires, with response rates is shown in Table II.

Not all subjects gave usable answers to every question. For example, the response rates to the questions on milk consumption were 71%, 63% and 57%.

In addition there were replies from 78 mothers of cases (60.5% of responding sons), 133 mothers of cancer controls (61.6% of responding sons) and 125 mothers of population controls (67.6% of responding sons).

The dates of birth were matched within 2.5 years but there was a bias towards older subjects in population controls and the average age of cases was 0.7 years less than cancer controls and 1.7 years less than population controls. The median year of diagnosis of responding cases was 1987–8, and of cancer controls 1987. Height and weight were not significantly different in the three groups. Social class was higher in population controls but not significantly so.

The average age of testicular cancer patients was 43 years on the notional day of the study (1 September 1992) and the mean age at diagnosis was 36.2 (range 15–65). Some testicular cancer cases who were eligible for the study were not included because a matching cancer control could not be found. Because this was more likely to occur with younger cases, the mean age at diagnosis of cases entered was higher than the mean age at diagnosis of eligible cases (35.8), (after weighting their average age in proportion to the numbers actually selected for each year of diagnosis).

### *Consumption of milk, dairy products and other food (Tables III–V)*

The estimates of mean consumption of milk, dairy products, fruit and vegetables at the age of 17 are shown in Table III. For comparison, the present consumption of milk is also shown. The only notable difference between groups was in milk drinking; cases drinking 1.00 pints per day, cancer controls 0.94 pints per day, which was significantly more than population controls, who drank 0.80 pints per day. Although cases consumed more than cancer controls, this difference was not significant. Present consumption of milk shows a similar pattern, although there was in this case only a very small difference between cases and cancer controls.

When the subjects were stratified by milk consumption at 17, there was a clear gradient with the testicular cancer patients having most high-consumers and population controls having the least (Table IV). The distribution is not skewed by a minority of high consumers, the one outlier being a cancer control.

**Table I** Characteristics of the subjects and response rates

| | *Cases* | *Cancer controls* | *Population controls* |
|---|---|---|---|
| Number | 129 | 211 | 184 |
| Response rate (%) | 73.0 | 65.0 | 57.0 |
| Mean age (years) | 42.7 | 43.4 | 44.4 |
| Mean height (cm) | 177.7 | 177.1 | 178.3 |
| Mean weight (kg) | 71.6 | 69.6 | 70.7 |
| Mean social class[a] | 3.18 | 3.16 | 2.90 |
| Number (%) with undescended testis[b] | 18 (14.4) | 5 (2.44) | 5 (2.73) |
| Hare lip or cleft palate | 0 | 0 | 0 |

[a]Excluding 37 subjects (seven cases, 23 cancer controls and 12 population controls) retired or unemployed. [b]Non-response has been taken to be negative.

**Table II** The number of subjects who received questionnaires, with response rates

| | n | *Replies* | *Response rate (%)* |
|---|---|---|---|
| Cases | 177 | 129 | 73 |
| Cancer controls | 333 | 216 | 65 |
| Population controls | 322 | 185 | 57 |

**Table III** Estimated mean consumption of milk, dairy products, fruit and vegetables in adolescence (standard deviation)

| *[a]Number of subjects (range)* | *Cases (114–126)* | *Cancer controls (175–211)* | *Population controls (168–184)* |
|---|---|---|---|
| Milk (pints $day^{-1}$) | 1.00 (0.51) | 0.94 (0.56) | 0.80 (0.44) |
| Cream (times $week^{-1}$) | 0.47 (0.85) | 0.36 (0.54) | 0.39 (0.58) |
| Yoghurt (times $week^{-1}$) | 1.32 (2.34) | 1.13 (1.98) | 1.24 (1.94) |
| Cheese (times $week^{-1}$) | 3.67 (3.01) | 3.31 (2.58) | 3.57 (2.80) |
| Apples (times $week^{-1}$) | 2.68 (2.97) | 2.66 (2.73) | 3.03 (2.98) |
| Oranges (times $week^{-1}$) | 0.96 (1.35) | 0.84 (1.28) | 1.28 (2.12) |
| Vegetable salad (times $week^{-1}$) | 2.46 (2.51) | 2.21 (2.24) | 2.57 (2.27) |
| Fruit salad (times $week^{-1}$) | 0.41 (0.51) | 0.47 (0.71) | 0.54 (0.87) |
| Present milk consumption | 0.73 (0.34) | 0.72 (0.42) | 0.63 (0.35) |

[a]Number of subjects varied for the different dietary items, but details of milk were provided by almost all controls.

As expected, more cases had a history of UDT, and if we assume non-response or a response of 'don't know' to the question about UDT is equivalent to 'no', then the relative risk of UDT between cases and all controls was 6.46 (95% CI 2.74–15.19).

The results of logistic regression analysis are shown in Table V. The variables shown were selected because they had an appreciable effect, even if not statistically significant, or because previous studies had suggested their relevance. Because the actual difference in milk consumption was small (about 0.2 pints between cases and population controls), the odds ratio was expressed in relation to quarter pints.

If any or all other variables were included, there was no appreciable alteration to the results shown. The results of multivariate analysis confirm the increased likelihood of UDT in cases, an effect of height, even allowing for weight and social class, albeit usually non-significant, and the effect of milk consumption at 17. The effect of the consumption of other dairy products virtually disappeared when other variables were taken into account.

Increasing milk consumption increased the probability of being a case or cancer control significantly but there was no significant difference in risk between them.

There was a tendency for cases to consume fewer apples, oranges and vegetable and fruit salads than the population controls, but more than the cancer controls. These differences were not significant and were possibly due to a social class effect, since 63% of population controls had non-manual occupations, compared with 54% of cases (unemployed not included) and the effect of these differences was almost completely lost when the fruit and vegetable consumption was included in the multivariate analysis.

**Table IV** Numbers of cases and controls (per cent of group) by category of milk consumption

| *Consumption* | *Cases* | *Cancer controls* | *Population controls* | *Total* |
|---|---|---|---|---|
| Less than 0.5 pints | 15 (12.0) | 34 (16.3)<br>11 (12.2)(NE)[a]<br>23 (19.3)(E) | 42 (23.0) | 91 (17.6) |
| 0.5 | 55 (44.0) | 96 (45.9)<br>39 (43.3)(NE)<br>57 (47.9)(E) | 91 (49.7) | 242 (46.8) |
| 1.0 | 34 (27.0) | 44 (21.1)<br>24 (26.7)(NE)<br>20 (16.8)(E) | 31 (16.9) | 109 (21.1) |
| 1.5 or more | 21 (16.8) | 35 (16.7)<br>16 (17.8)(NE)<br>19 (16.0)(E) | 19 (10.4) | 75 (14.5) |

Analysis using $\chi^2$ test for trend: cases *vs* population controls, $\chi^2=9.51, p=0.0027$; cases *vs* cancer controls, $\chi^2=0.75, p=0.386$; cases *vs* all controls, $\chi^2=4.43$, $p=0.035$; cancer controls *vs* population controls $\chi^2=6.16$, $p=0.013$; cases and non-epithelial (NE) cancer controls *vs* epithelial (E) cancer controls and population controls, $\chi^2=10.7, p=0.0011$. [a]See discussion.

### Discussion

The hypothesis being tested, that men who developed testicular cancer had consumed more milk in adolescence, was supported by the evidence.

### *The scope for bias*

However, the response rate was lower than we would have liked, particularly among population controls. We therefore tried to estimate the degree to which this could have introduced bias. The responding population controls included more non-manual workers but, as the difference between the milk consumption between men with manual and non-manual occupations was negligible (0.02 pints), this potential bias could not explain the observed difference.

Cases included more respondents who estimated they had consumed 'more' milk in adolescence (56% *vs* 50% *vs* 49%). If the proportion reporting 'more' in cases is reduced to the level of the control groups, the estimated consumption of milk in adolescence is reduced by a maximum 0.04 pints.

The experience of cancer may itself affect recall, and for this reason, cancer controls were selected as well as population controls. As the consumption of milk was similar in cancer cases and controls, this might be an explanation of the results. We noted, however, that in this age group many of the cancer controls were non-epithelial cancer, mainly lymphomas and leukaemias. As Ursin *et al.* (1990) found a strongly positive relationship between milk consumption and tumours of the lymphatic system, it seemed possible that cancer controls did not have a homogenous relationship to milk consumption. We, therefore, divided the cancer controls into those with non-epithelial cancers (Hodgkin's disease, non-Hodgkin's lymphoma, leukaemias, tumours of the brain, connective tissue and bone) and epithelial tumours dominated by colorectal cancers and melanomas.

Table IV presents a subset of these data by category of milk consumption with cancer controls divided into two groups, and as would be expected from the average consumption, there is a gradient with a higher proportion of high milk consumers in cancer cases and in cancer controls with non-epithelial cancers.

This is an interesting result in itself, but it does suggest that recall bias would be unlikely to explain the result by affecting patients with non-epithelial cancer preferentially.

All subjects were alive and mortality is low in testicular cancer. If the testicular cancer patients excluded by death had never drunk milk this would only account for half the difference in consumption between testicular cancer patients and population controls.

**Table V** Multivariate analysis comparing cases, cancer controls and population controls for six variables

| | *Cases vs all controls* | | *Cases vs population controls* | | *Cases vs cancer controls* | | *Cancer controls vs population controls* | |
|---|---|---|---|---|---|---|---|---|
| | *OR*[a] | *95% CI* | *OR*[a] | *95% CI* | *OR*[a] | *95% CI* | *OR*[a] | *95% CI* |
| Undescended testis | 6.46 | 2.74–15.19*** | 7.19 | 2.36–21.90*** | 5.94 | 2.07–17.12*** | 1.21 | 0.40–3.60 |
| Age | 0.99 | 0.97–1.02 | 0.99 | 0.96–1.05 | 1.00 | 0.97–1.03 | 0.98 | 0.96–1.01 |
| Social class[b] | 1.12 | 0.92–1.35 | 1.18 | 0.95–1.46 | 1.04 | 0.84–1.29 | 1.14 | 0.94–1.37 |
| Height | 0.99 | 0.88–1.08 | 0.92 | 0.82–1.03 | 1.03 | 0.92–1.15 | 0.89 | 0.81–0.98* |
| Weight | 0.98 | 0.99–1.02 | 1.00 | 0.99–1.02 | 1.00 | 0.99–1.02 | 1.00 | 0.99–1.01 |
| Milk consumption at 17 | 1.23 | 1.09–1.38*** | 1.39 | 1.19–1.63*** | 1.14 | 1.00–4.04 | 1.15 | 1.01–1.30* |

[a]The odds ratio (OR) is expressed as the charged risk of being in the first category (cases etc.) with each extra unit of the variable concerned. Units are: height, inches: milk consumption, 1/4 pints; weight, pounds; age, years. [b]Social class, Registrar General's social class where 1 = I, 2 = II, 3 = III non-manual, 4 = III manual, 5 = IV, 6 = V. Thirty seven retired and unemployed not included (7.6% of all subjects). *$P<0.05$, **$P<0.01$, ***$P<0.001$.

It is axiomatic that if the whole population is exposed to a risk factor, then it is very difficult to identify it. If this is the case, the alternative is to correlate incidence of disease with levels of exposure. In England over the past half century, most people have consumed milk, and in this study, there is an apparent association between the level of milk consumption and the incidence of non-epithelial cancers. There is also a correlation between national dairy product consumption and the incidence of testicular cancer (Food and Agriculture Statistics, 1971; Muir *et al.*, 1987).

In England and Wales, the incidence of testicular cancer has been rising in recent decades while milk consumption has been falling. The modal calendar years of diagnosis in this study are 1987–8, and the average age 43. Cases would therefore have been about age 16 in 1965, and national milk consumption reached a peak between 1960 and 1968. Thus, even if milk were the only risk factor, given a 15–20 year latent period, it would be quite possible to reconcile a recently rising incidence rate with falling consumption.

However, even if milk was causally related to the development of non-epithelial cancer, it would only be one of possibly many promoting factors, and the change in incidence might be the result of changes in an initiating factor acting during gestation. It is also possible that milk consumption is not causal but is a confounder for another factor.

### Conclusion

The hypothesis based on national food consumption patterns suggests that patients with testicular cancer may have consumed more milk in adolescence than the general population. This turned out to be the case, although the scope for bias remained uncomfortably large. Patients with non-epithelial cancers had a similar milk consumption to those with testicular cancer, suggesting that testicular cancer is not unique. Milk may either be a promoting factor or a marker of other aspects of lifestyle that stimulate testicular and other non-epithelial cancers in young men.

**Acknowledgements**
The study was supported by the Harnett Fund of the University of Cambridge. Particular thanks are due to Sue Worden who administered the survey, the East Anglian Reporting System of the Royal College of General Practitioners and all the General practitioners who took part, Jane Wright of the East Anglian Cancer Registry, Dr Sheila Bingham of the Dunn Nutrition Unit for valuable advice on dietary questionnaires and, of course, the subjects and their mothers.

### References

ARMSTRONG B AND DOLL R. (1975). Environmental factors and cancer incidence and mortality in different countries, with special reference to dietary practices. *Int. J. Cancer*, **15**, 617–631.

CHENG KK, DAY NE, DUFFY SW, LAM TH, FOK M AND WONG J. (1992). Pickled vegetables in the aetiology of oesophageal cancer in Hong Kong Chinese. *Lancet*, **339**, 1314–1318.

FOOD AND AGRICULTURE ORGANIZATION OF THE UN. (1971). *Production Year Book 1970*. Food and Agriculture Organization of the UN: Rome.

GIOVANNUCCI E, STAMPFER MJ, COLDITZ GA, RIMM EB, TRICHOPOULOS D, ROSNER BA, SPEIZER FE AND WILLETT WC. (1993). Folate, methionine and alcohol intake and risk of colorectal adenoma. *J. Natl Cancer Inst.*, **85**, 875–884.

MUIR C, WATERHOUSE J, MACK T, POWELL J AND WHENAL S. (1987). *Cancer Incidence in Five Continents*. International Agency for Research on Cancer: Lyon.

STEARNS EL, MACDONNELL JA, KAUFMAN BJ, PADUA R, LUCHAN TS, WINTER JSD AND FAIMAN C. (1974). Declining testicular function with age. *Am. J. Med.*, **57**, 761–766.

URSIN G, BJELKE E, HEUCH I AND VOLLSET SE. (1990). Milk consumption and cancer incidence: a Norwegian prospective study. *Br. J. Cancer*, **61**, 456–459.

VERMEULEN A, VANDEWEGHE M, RUBENS R, COMMAIRE F, VERDONCK L AND DHONT M. (1974). *Recent Advances in Reproductive Endocrinology*. Academic Press: London.